# Accelerometer-measured physical activity levels in children and adolescents with autism spectrum disorder: A systematic review

**DOI:** 10.1016/j.pmedr.2020.101147

**Published:** 2020-06-18

**Authors:** Xiao Liang, Ru Li, Stephen H.S. Wong, Raymond K.W. Sum, Cindy H.P. Sit

**Affiliations:** a Department of Sports Science and Physical Education, The Chinese University of Hong Kong, Hong Kong; b Physical Education Unit, Shenzhen University, China

**Keywords:** Physical activity guidelines, Autism spectrum disorders, Schools, Socio-ecological model

## Abstract

Associations between physical activity (PA) and health benefits are well documented. Evidence indicates that children and adolescents with autism spectrum disorder (ASD) are less physically active than their typically developing peers. The purpose of this systematic review is to provide a comprehensive summary of the PA levels of children and adolescents with ASD and the associated factors that affect their PA levels by applying a socio-ecological model (SEM). Seven databases (PubMed, CINAHL, SPORTDiscus with Full Text, MEDLINE, EMBASE, ERIC, and PsychINFO) were searched in June 2019 to identify studies examining accelerometer-measured PA and factors affecting the PA levels of children and adolescents with ASD, aged 6–17 years. Two researchers independently screened studies, assessed methodological quality, and summarized relevant data. Twenty-one studies were included in the detailed review. Only 42% of the participants met the PA guidelines (i.e., children and adolescents aged 5–17 years should do at least 60 min of moderate to vigorous PA daily). By applying the SEM, multi-level factors ranging from intrapersonal to community levels that positively or negatively influenced PA levels in children and adolescents with ASD were identified. This review indicates that children and adolescents with ASD have low PA levels, and that there are multi-level factors that affect their PA. There is a pressing need to design effective PA interventions that promote activity accrual in the school, family, and community settings for children and adolescents with ASD.

## Highlights

• Only 42% of them met the minimum WHO PA guideline.
• Younger age and better fitness, motivation and sleep were associated with higher PA.
• HH Having support from peers with TD, teachers and family were related to increased PA.
• Unprepared PE and lacking equipment were associated with decreased PA at school.
• Lack of community-based PA opportunities during leisure time were related to low PA.
• Policies or laws were not associated with PA.

## 1 Introduction

Regular participation in physical activity (PA) plays a crucial role in promoting and maintaining a life-long healthy lifestyle for all ages and abilities. The physical and psychological benefits of PA for children and adolescents with disabilities are well documented. They include (1) maintaining normal muscle strength, flexibility, and function (Martin Ginis et al., 2016); (2) improving health and physical fitness (Collins and Staples, 2017); (3) enhancing confidence and self-efficacy (Shields et al., 2012, Must et al., 2015); and (4) acquiring social support from typically developing (TD) peers (Pan et al., 2015, Pan et al., 2011b). Compared to their TD peers, children and adolescents with disabilities tend to be more sedentary and experience more secondary or chronic diseases such as obesity (Centers for Disease Control and Prevention, 2010). It was recommended that, regardless of any disability, children and adolescents aged 6–17 years should engage in 60-minutes or more of moderate-to-vigorous physical activity (MVPA), on a daily basis, to achieve health benefits (U.S. Department of Health and Human Services, 2018, World Health Organization, 2010).

Autism spectrum disorder (ASD) is a general developmental disorder that affects an individual’s social communication and behaviour and intellectual development (American Psychiatric Association, 2016). The current prevalence of ASD is 1.5% of the global population (Baxter et al., 2015, Lyall et al., 2017). In addition to a lack of social skills, previous studies have indicated that being overweight, suffering from obesity, and inactivity occur at higher rates among children with ASD than among their TD peers (Pan and Frey, 2006, Curtin et al., 2010, Menear and Neumeier, 2015).

Despite the evidence to support the PA benefits for a healthy lifestyle, the findings from the 2017 Dutch Report Card+ (Burghard et al., 2018) and the 2018 Finland Report Card (Kämppi et al., 2018) showed that only 26% of Dutch children and adolescents with a disability or chronic disease and 14% of Finnish adolescents with disabilities met the recommended PA guideline (PAG), respectively.

Schools are important settings for promoting PA in children and adolescents, and it was recommended that children and adolescents should spend at least 50% of their class time engaging in MVPA during physical education class (Centers for Disease Control and Prevention, 2010), and 40% of their recess time on MVPA (Ridgers et al., 2005). Previous studies have examined the PA levels of children and adolescents with and without ASD in schools. For example, Pan et al. (2011a) found that adolescents with ASD (36.73%) and their TD peers (44.63%) did not meet the PAG for physical education lessons. Meanwhile, there were no significant differences in the time spent on MVPA during recess time between children with (27.7%) and without ASD (36.15%) (Pan, 2008b), and both groups failed to meet the PAG for recess.

**Fig. 1:**
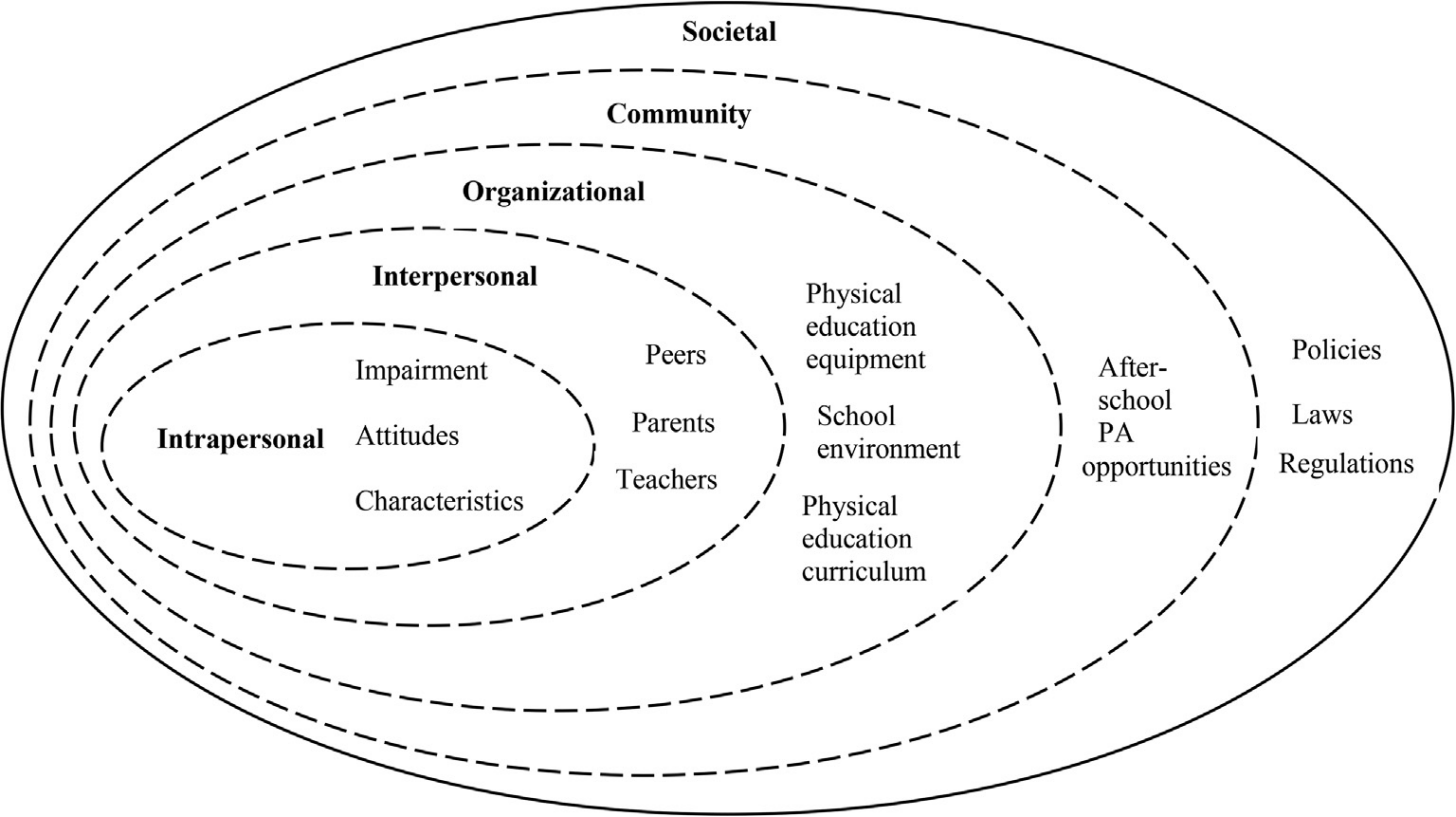
Socio-ecological model. Adapted from McLeroy et al. (1988).

The issues that affect the PA levels in children and adolescents with ASD are complex. The socio-ecological model (SEM) has been used to identify the inhibitors and facilitators for the PA levels of individuals with disabilities (e.g., Obrusnikova and Cavalier, 2011, Úbeda-Colomer et al., 2019, Vasudevan et al., 2015). The SEM depicts five levels of influence (see Fig. 1). The first level (centre of the model) involves intrapersonal factors, such as impairment, age, gender, attitude, and knowledge. The second level focuses on interpersonal factors, which include social relationships with teachers, peers, and family members. The third level includes organisational factors, such as physical education courses, in-school PA programmes, and the available PA facilities and equipment. The fourth level centres on community factors, such as public transportation, community-based PA programmes, and the public environment. The fifth level (outermost level of the SEM) is societal, and includes public policies, laws, and regulations at various levels.

To our knowledge, no systematic reviews have yet examined the accelerometer-measured PA levels of children and adolescents with ASD and the associated factors that affect their PA levels. More importantly, no systematic reviews have taken the PAG into consideration for this population group. A lone review covering children with ASD aged 0–18 years old (Jones et al., 2017) reported the prevalence and correlates of PA, but did not consider PAG or apply any theoretical frameworks. Thus, this systematic review had two main aims: (1) to examine the published literature to determine the PA levels of children and adolescents with ASD and whether they met different PAGs; and (2) to identify the factors that affected the PA levels in children and adolescents with ASD at different levels by using the SEM as a theoretical framework.

## 2 Methods

### 2.1 Search strategy

A systematic search of the relevant literature was conducted using seven electronic databases: PubMed, CINAHL, SPORTDiscus with Full Text, MEDLINE, EMBASE, ERIC, PsychINFO. The search was limited to “English,” “human-related,” and “peer-reviewed” articles. The initial search was undertaken using the following four key terms: physical activity, physical activity levels, ASD, children or adolescents. The search key words for each main term were developed from the search strategies of previous reviews related to PA and children or adolescents with ASD and expert opinions in the fields of PA and special education. In each database, each main term and associated synonyms were identified on the basis of the following paradigm: “([Physical activit* OR Leisure activit* OR Exercise* OR Sport* Participation OR Motor activities OR Physical education OR Physical exercise OR Fitness OR Exercise therapy]) AND ([Physical activity level OR Physical activity measurement OR Motor activity level OR Motor activity measurement]) AND ([Autism Spectrum Disorder OR Autism* OR ASD OR Autistic* OR Asperger Syndrome* OR Pervasive Developmental Disorder OR PDD OR PDDNOS]) OR ([Children OR Child OR Childhood OR Adolescent OR Youth OR Teenager])”. In addition, two independent reviewers also conducted a manual search to identify relevant articles.

### 2.2 Inclusion and exclusion criteria

Studies were included if they:

1. quantitatively measured the PA levels of children and adolescents with ASD;
2. were observational research (i.e., cross-sectional, case control, and cohort);
3. reported the PA levels in the form of duration in minutes or in any environment (e.g., recess, physical education class, after school);
4. were peer-reviewed articles with full-text available written in English;
5. included participants aged 5–17 years; and
6. provided complete research data where the length of MVPA could be computed.

Studies were excluded if they:

1. were written in a language other than English;
2. were intervention research (e.g., clinical and field trials);
3. included participants with other types of disabilities where the data specific to children and adolescents with ASD could not be differentiated; and
4. were review studies, case/government reports, conference papers, book chapters, or policy documents.

### 2.3 Data selection

**Fig.2:**
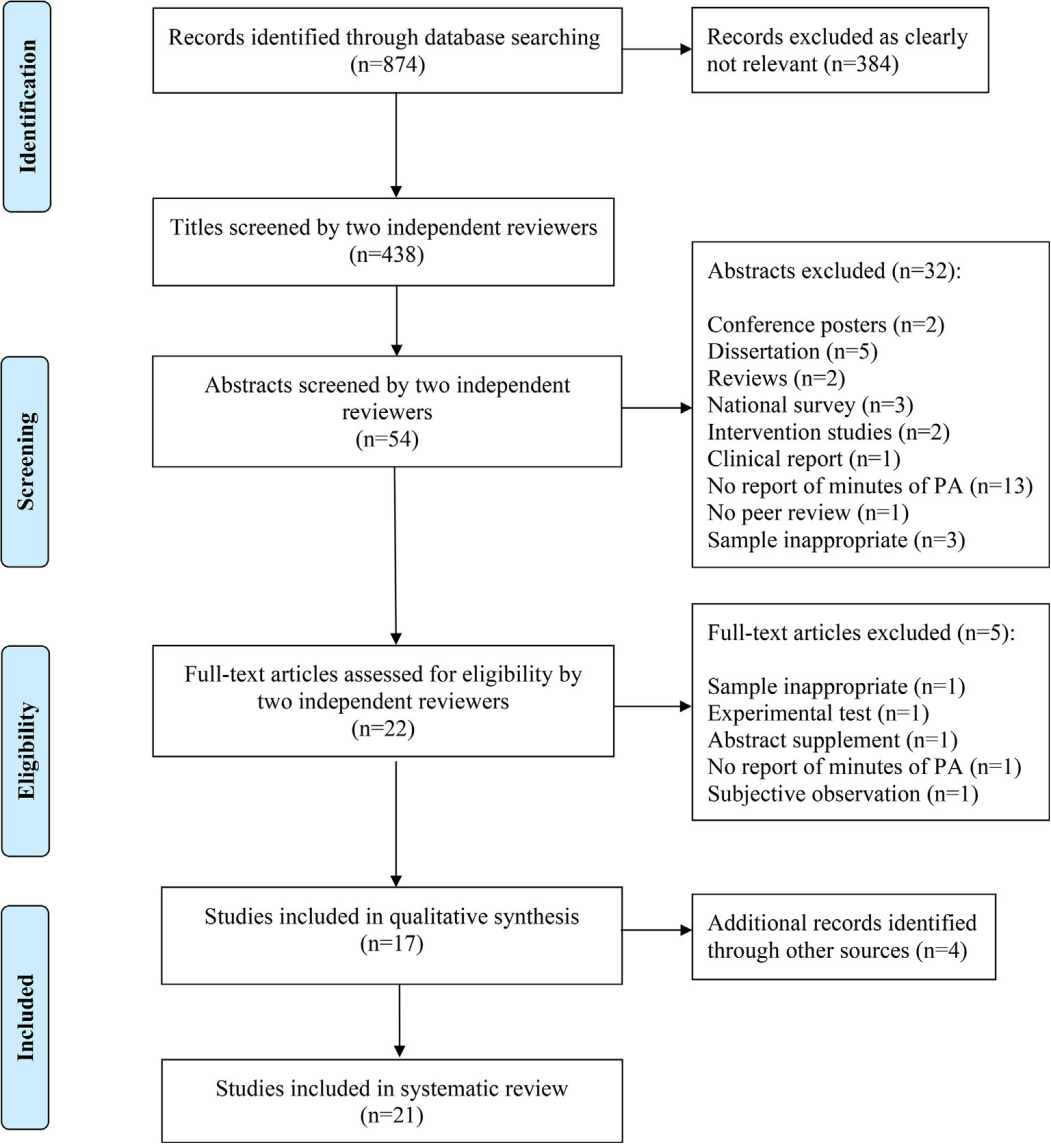
Flow diagram showing the study selection process (adapted from the PRISMA process, Liberati et al., 2009).

A total of 874 articles were found in the initial search of the seven databases described. Fig. 2 illustrates the numbers of articles screened and those that met the inclusion criteria. To ensure the accuracy of the systematic search process, two reviewers who were familiar with the field of disability and PA research conducted a multi-step search process and screened the titles, abstracts, and full-length texts to make an initial assessment independently. If the two reviewers had any disagreements, a third reviewer discussed the paper with the two reviewers and made a final decision. Fifty-four abstracts met the inclusion criteria, with an inter-rater reliability of Kappa value = 0.88 between the two reviewers. After screening the abstracts, 22 articles were selected to conduct full-text screening. Furthermore, 17 articles passed the inclusion criteria with an inter-rater reliability of Kappa value = 0.94. In addition, four manually searched articles agreed on by the two reviewers met the inclusion criteria. Finally, 21 articles were selected for the systematic review.

### 2.4 Quality assessment

The McMaster Critical Reviewer Form for quantitative studies (Law et al., 1998) was used to evaluate the methodological quality of the included articles, based on the Guidelines for Critical Review Form-Quantitative Studies (Law et al., 1998). The numerical rating criteria for non-experimental quantitative studies developed by Imms (2008) were also employed to interpret the methodological quality. These scoring criteria have been widely used in previous systematic reviews related to disability and PA research (Shields et al., 2012, Bloemen et al., 2015, Li et al., 2016). The three key criteria in the included studies were evaluated in the present study: sample, measurement, and analyses (Li et al., 2016). *Sample* evaluated whether the selection bias was reduced (e.g., representative of selected population), whether the sample size was suitable for the research design and questions, and whether the characteristics of participants were clearly described by the authors. *Measurement* examined whether the measurement bias was reduced (e.g., reliability and validity of the measurement tool, recall/memory). *Analyses* examined whether reported analyses were appropriate for the research questions and outcome measures (e.g., reported statistical significance, point estimates, provided variability, and discussed clinical importance) (Shields et al., 2012, Bloemen et al., 2015, Li et al., 2016). Each criterion was scored either with one star, meaning no evidence shows that the study can meet any criteria; or two stars, indicating that certain pieces of evidence in the study can meet the criteria, or that the report is unclear; or three stars, indicating that the evidence in the study completely meets the criteria (Imms, 2008, Shields et al., 2012, Bloemen et al., 2015, Li et al., 2016). Two reviewers independently evaluated the methodological quality of the studies selected for inclusion. Discrepancies between the two reviewers were discussed until a consensus was reached. If the two reviewers were unable to agree, a third researcher made a final decision after a discussion with the two reviewers.

### 2.5 Data extraction

Data were extracted using a standardized form based on previous studies (Dairo et al., 2016, Leung et al., 2017, Li et al., 2016), including relevant data about bibliographic details (author and year), participant characteristics (sample size, gender, age range, school placement, location, classification of ASD severity), study design, measures of PA, percentage of participants meeting the PAG, percentage of time spent in MVPA at different school settings, and PA-related factors by applying the SEM.

### 2.6 Data analysis

For the PA levels of children and adolescents with ASD, their MVPA was calculated to actual minutes and percentage on school days and weekends, based on the cut-off point standardized by the global recommended PAG. Specifically, the duration and percentage of MVPA on school days were analyzed in four settings (overall, physical education class, recess, and after-school time). MVPA spent at weekends was also calculated. To identify factors as being either “related” or “not related” to the PA levels of children with ASD, the potential factors showing a significant association with the PA levels were summarised.

## 3 Results

### 3.1 Descriptive characteristics of included studies

Table 1 summarizes the sample size, gender, age range, school placement, study location, classification of ASD severity, and the rating of the included studies. More than half (52%) of the studies described in the 21 included papers were conducted in the USA, and 43% were conducted in Taiwan. The study sample size ranged from 6 to 72, and 88% of the participants were male students, with a mean age ranging from 8.2 to 15.0 years. The participants were mostly from three particular types of school: mainstream schools (57%), special schools (24%), and home schools (14%). Thirteen studies (62%) provided a clear classification of ASD severity. For the ratings on the quality assessment criteria, the inter-rater reliability between the two researchers was calculated, for Sample (k = 0.83), Measurement (k = 0.87) and Analyses (k = 0.77). No study received three stars for all criteria, but some studies obtained three stars for one or two criteria, and most studies scored two stars for all three criteria. For the *Sample* component, all studies used a convenience sample. Ten studies recruited over 30 participants, but two studies only had 6 participants. Thirteen studies gave detailed participant characteristics. For the *Measurement* component, seven studies used objective-measured PA with questionnaires or observation, and were given three stars because they were able to promote the understanding of PA-related factors behind PA levels. For the *Analyses* component, 8 studies were given three stars because they fully reported results in terms of statistical significance and gave a clear description of limitations and implications.

**Table 1:** Summary of participants’ characteristics of included studies.

Author & Year	Sample Size (ASD)	Gender	Age Range (Mean, SD)	School Placement	Location	Classification of ASD Severity	Quality Criteria		
							Sample	Methods	Analysis
Pan and Frey (2005)	30	27M, 3F	10–19 (13.2, 2.1)	SS, MS, HS	USA	Autism (14), Asperger syndrome (12), PDDNOS (4)	**	***	**
Sandt and Frey (2005)	15	10M, 5F	5–12 (9.5, 1.9)	MS	USA	Autism (9), Asperger syndrome (2), PDDNOS (4)	**	***	***
Pan and Frey (2006)	30	27M, 3F	10–19 (13.2, 2.1)	SS, MS, HS	USA	Autism (14), Asperger syndrome (12), PDDNOS (4)	**	***	**
Pan (2008a)	24	23M, 1F	7–12 (9.2, 1.4)	MS (P)	Taiwan	Autism (21), Asperger syndrome (3)	**	**	***
Pan (2008b)	24	23M, 1F	7–12 (9.2, 1.4)	MS (P)	Taiwan	Mild or high-functioning Autism (12), moderate Autism (9); Asperger syndrome (3)	**	**	***
Pan (2009)	25	25M	7–12 (9.28, 1.46)	MS (P)	Taiwan	Mild or high-functioning Autism (11), moderate Autism (8); Asperger syndrome (6)	**	**	**
Obrusnikova and Cavalier (2011)	14	12M, 2F	8–14 (10.64, 1.65)	SS, MS, HS	USA	Autism (1), Asperger syndrome (10), PDDNOS (3)	**	***	***
MacDonald et al (2011)	72	55M, 17F	9–18 (?, ?)	NR	USA	NR	*	**	*
Pan et al (2011a)	19	19M	? (14.19, 0.82)	MS (S)	Taiwan	NR	*	**	*
Pan et al (2011b)	25	25M	? (14.26, 0.89)	MS (S)	Taiwan	Mild Autism (15), Asperger syndrome (10)	**	**	**
Pan et al (2011c)	35	35M	7–12 (9.65, 0.54)	MS (P)	Taiwan	Mild autism (22), Asperger syndrome (13)	**	**	***
Bandini et al (2012)	53	44M, 9F	3–11 (6.6, 2.1)	NR	USA	NR	*	***	***
Tyler et al (2014)	17	9M, 8F	9–17 (12.6, 2.3)	NR	USA	NR	*	*	*
Ayvazoglu et al (2015)	6	4M, 2F	4–13 (7.5, 3.1)	NR	USA	High-functioning Autism (2), Autism (1); Asperger syndrome (2); PDDNOS (1)	*	***	*
Pan et al (2015)	30	30M	12–17 (14.51, 1.54)	MS (S)	Taiwan	Mild autism (23), Asperger syndrome (7)	**	**	**
Wachob and Lorenzi (2015)	10	9M, 1F	9–16 (11.8, 2.3)	NR	USA	NR	*	**	*
Pan et al (2016)	35	35M	12–17 (14.55, 1.54)	MS (S)	Taiwan	Mild autism (25), Asperger syndrome (10)	**	**	***
Stanish et al (2017)	35	29M, 6F	13–21 (15.9, 1.7)	SS	USA	NR	**	***	**
Haegele et al (2018)	6	4M, 2F	8–16 (12.4, ?)	NR	USA	NR	*	**	**
Chu et al (2019)	63	63M	12–18 (?, ?)	NR	Taiwan	Mild autism (20), Asperger syndrome (43)	**	**	**
Garcia-Pastor et al. (2019)	44	33M, 11F	7–18 (12.31, 1.67)	SS	Spain	NR	**	**	***

Note: M = male; F = female; SS = special school; MS = mainstream school(S = secondary school & P = primary school); HS = home school; SD = standard deviation; NR = not reported; PDDNOS = Pervasive Developmental Disorder-Not Otherwise Specified; ?=no data provided; *=no criteria were met within that component; **=only some criteria were met within component; ***=all criteria were met within that component.

Table 2 summarises the study design, measures of PA, percentage of participants meeting PAG, and time spent on MVPA in the included studies. In addition to the use of accelerometers, one study (4.8%) used both accelerometers and direct observation (Sandt and Frey, 2005), and six studies (28%) used accelerometers alongside questionnaires (Pan and Frey, 2006, Pan et al., 2011a, Obrusnikova and Cavalier, 2011, Bandini et al., 2012, Ayvazoglu et al., 2015, Stanish et al., 2017).

**Table 2:** Summary of included studies on the PA levels of and PA related findings.

Author & Year	Study Design	Measures of PA	PAG	% of Participants Meeting PAG	Time Spent in Mins (MVPA%) at Different PA Settings					
					Weekday				Weekend	Wear time
					Overall	Physical Education	Recess	After-school		
Pan and Frey (2005)	Cross-sectional	Accelerometer & Questionnaire	a	47^a^	NR	NR	NR	NR	NR	78.2^a^
Sandt and Frey (2005)	Cross-sectional	Accelerometer & Observation (BEACHES)	a	67^a^	127.5 ± 72.3^a^ (24%)	12.8 ± 6.8 (41%)	15.5 ± 8.8 (58%)	51.9 ± 35.7 (30%)	NR	NR
Pan and Frey (2006)	Cross-sectional	Accelerometer & Questionnaire (Child/Adolescent Activity Log)	a	47^a^	NR	NR	NR	NR	NR	82.48^a^
Pan (2008a)	Cross-sectional	Accelerometer	b c	30.83^b^	NR	18.5 ± 19.25 (46%)^b^	27.63 ± 8.8 (28%)^c^	NR	NR	NR
Pan (2008b)	Cross-sectional	Accelerometer	c	0^c^	NR	NR	27.58 (28%)^c^	NR	NR	NR
Pan (2009)	Cross-sectional	Accelerometer	b c	NR	NR	18.7 (55%)^b^	23.51 (47%)^c^	NR	NR	NR
Obrusnikova and Cavalier (2011)	Cross-sectional	Accelerometer & Photo-voice	a	21^a^	NR	NR	NR	NR	NR	81.7 ^a^ (6%)
MacDonald et al (2011)	Cross-sectional	Accelerometer	a	100^a^	41.67^a^ (4.9%)	NR	NR	13.8 (12%)	NR	110.8^a^ (11%)
Pan et al (2011a)	Cross-sectional	Accelerometer	b	NR	NR	14.88 (37%)^b^	NR	NR	NR	NR
Pan et al (2011b)	Cross-sectional	Accelerometer	b	NR	NR	13.38 (33%)^b^	NR	NR	NR	NR
Pan et al (2011c)	Cross-sectional	Accelerometer	a b c	100 & 71.4^a^ (weekdays& weekends)	104^a^ (13%)	11.38 (31%)^b^	3.96 (32%)^c^	36.39 (14%)	80.58^a^ (11%)	NR
Bandini et al (2012)	Cross-sectional	Accelerometer & PA Checklist	a	23^a^	48^a^ (6.3%)	NR	NR	NR	53.5^a^ (7.4%)	NR
Tyler et al (2014)	Cross-sectional	Accelerometer	a	100^a^	NR	NR	NR	NR	NR	165.9^a^ (19%)
Ayvazoglu et al (2015)	Cross-sectional	Accelerometer & Q-sort	a	0^a^	NR	NR	NR	NR	NR	34.33^a^
Pan et al (2015)	Cross-sectional	Accelerometer	a b c	46.7^a^ 6.7^b^ 16.67^c^	69.61^a^ (8%)	14.34 (31%)^b^	2.32 (23%)^c^	24.24 (20%)	NR	NR
Wachob and Lorenzi (2015)	Cross-sectional	Accelerometer	a	50^a^	73.36^a^	NR	NR	? (13%)	63.27^a^	NR
Pan et al (2016)	Cross-sectional	Accelerometer	a	37^a^	64.23^a^ (7.9%)	NR	NR	NR	63.14^a^ (8.7%)	NR
Stanish et al (2017)	Cross-sectional	Accelerometer & Self-edited Questionnaire	a	14^a^	31^a^	NR	NR	NR	12.2^a^	25.6^a^
Haegele et al (2018)	Cross-sectional	Accelerometer	a	0^a^	13.28^a^ (2.4%)	NR	NR	NR	NR	NR
Chu et al (2019)	Cross-sectional	Accelerometer	a	44^a^	NR	NR	NR	NR	NR	65.15^a^ (8.38%)
Garcia-Pastor et al. (2019)	Cross-sectional	Accelerometer	NR	NR	65.9	NR	NR	NR	73.18	71.18

Note: PAG = physical activity guideline; BEACHES = Behaviours of Eating and Activity for Children’s Health: Evaluation System; OSRAC = Observational System for Recording Activity of Children; NR = not reported; ? = no data provided; MVPA = moderate to vigorous physical activity; a = children and adolescents should spend at least 60 mins on MVPA daily; b = children and adolescents should use 50% of physical education class time for MVPA; c = children and adolescents should use 40% of recess time for MVPA.

### 3.2 Physical activity levels under PAGs

Physical activity guideline (PAG) was cited as an outcome measure to evaluate the number of participants who met the PAG in question. Overall, 42% of participants met different PAGs; but only two studies reported that all participants met the PAG of 60-minutes’ daily MVPA (MacDonald et al., 2011, Tyler et al., 2014). Three studies (14%) indicated that no participants met the PAG (Pan, 2008b, Ayvazoglu et al., 2015, Haegele et al., 2018), whereas 10 studies (48%) revealed that less than 50% of participants met the PAG. Moreover, school settings accounted for the most time engaged in MVPA. Five studies (24%) indicated that participants only achieved the PAG of 60-min MVPA daily within the school environment (Sandt and Frey, 2005, Pan et al., 2015, Pan et al., 2011c, Wachob and Lorenzi, 2015, Pan et al., 2016). For physical education classes, seven papers (33%) reported that children and adolescents with ASD were inactive during physical education classes (Sandt and Frey, 2005, Pan, 2008a, Pan, 2009, Pan et al., 2015, Pan et al., 2011a, Pan et al., 2011b, Pan et al., 2011c). Participants in only one study met the PAG of 50% of class time engaged in MVPA (Pan, 2009). For recess, participants in two studies (9.5%) met the PAG of 40% of recess time doing MVPA (Sandt and Frey, 2005, Pan, 2009). Furthermore, participants in five studies spent an average of 17.8% of after-school time engaged in MVPA daily (Sandt and Frey, 2005, MacDonald et al., 2011, Pan et al., 2015, Pan et al., 2011c, Wachob and Lorenzi, 2015). Meanwhile five studies (Pan et al., 2011c, Bandini et al., 2012, Wachob and Lorenzi, 2015, Pan et al., 2016, Stanish et al., 2017) assessed the PA levels of children and adolescents with ASD on weekends, and three of them reported that participants met the PAG of 60-min MVPA (Pan et al., 2011c, Wachob and Lorenzi, 2015, Pan et al., 2016).

### 3.3 Time spent in MVPA at different settings

Table 2 shows the time spent in MVPA on weekdays and weekend days in different settings. On weekdays, 10 studies (48%) evaluated overall MVPA within school days ranging from 13.28 min to 127.5 min with a mean 56.95 min of MVPA per day (Sandt and Frey, 2005, MacDonald et al., 2011, Pan et al., 2011c, Bandini et al., 2012, Pan et al., 2015, Wachob and Lorenzi, 2015, Pan et al., 2016, Stanish et al., 2017, Haegele et al., 2018, Garcia-Pastor et al., 2019). During physical education class, six studies (29%) reported that children with ASD spent an average of 14.85 min on MVPA (Sandt and Frey, 2005, Pan, 2008a, Pan, 2009, Pan et al., 2015, Pan et al., 2011a, Pan et al., 2011b, Pan et al., 2011c). During recess, five studies (24%) found that children spent an average of 23.56 min on MVPA (Sandt and Frey, 2005, Pan, 2008a, Pan, 2009, Pan, 2008b, Pan et al., 2015, Pan et al., 2011c). After school, five studies (24%) reported that children participated in MVPA ranging from 13.8 min to 51.9 min (Sandt and Frey, 2005, MacDonald et al., 2011, Pan et al., 2015, Pan et al., 2011c, Wachob and Lorenzi, 2015). At weekends, four studies (19%) found that children spent over 60 min on MVPA (Pan et al., 2011c, Wachob and Lorenzi, 2015, Pan et al., 2016, Garcia-Pastor et al., 2019).

### 3.4 Factors affecting the PA levels of children and adolescents with ASD, using the SEM

Table 3 presents the factors that affected the PA levels of children and adolescents with ASD within the SEM.

**Table 3:** Summary of included studies on the PA related findings.

Author & Year	PA-related Factors in the Social Ecological Model				
	Individual	Interpersonal	Organisational	Community	Societal
Pan and Frey (2005)	Age -; Sedentary pursuits -	Parent support **00**	NR	After-school activity programs **00**	NR
Sandt and Frey (2005)	Sedentary pursuits -	NR	Enough PE time +; Limited recess time -	Unstructured home activities +	NR
Pan and Frey (2006)	Age -	NR	School time +; Limited recess time -	Types of PA programs & facilities **00**	NR
Pan (2008a)	NR	NR	Enough PE time +; Limited types of PA during recess -	NR	NR
Pan (2008b)	NR	NR	Long recess time +; Limited types of PA during recess -	NR	NR
Pan (2009)	Age +	Social support from PE teachers and peers +	NR	NR	NR
Obrusnikova and Cavalier (2011)	Sedentary pursuits -; Motivation to try different types of PA programs +	Lack of age-matched partners and support from parents -	Fruitful PE content +	Unsafe facilities -; Availability of PA equipment & programs +; Inconvenient transportation -	NR
MacDonald et al (2011)	Age -	NR	NR	Limited after-school PA patterns -	NR
Pan et al (2011a)	NR	Social initiations & interactions with peers +	PE content (fitness test, free play, outdoor PA) +	NR	NR
Pan et al (2011b)	Motivation (higher external regulation) +	NR	NR	NR	NR
Pan et al (2011c)	Age -	NR	Fewer PA opportunities (PE & after-school time) -	NR	NR
Bandini et al (2012)	NR	NR	NR	Fewer types of after-school PA -	NR
Tyler et al (2014)	Lower physical fitness (strength) -	NR	NR	NR	NR
Ayvazoglu et al (2015)	Lack of social skills -	Peer-related bullying -; Higher PA levels of parent **00**	NR	Fear of injury -	NR
Pan et al (2015)	NR	NR	Availability of in-school PA opportunities (PE & recess) +	NR	NR
Wachob and Lorenzi (2015)	Age -; Less WASO +; Lower sleep efficiency -	NR	NR	NR	NR
Pan et al (2016)	Higher physical fitness (cardiovascular endurance, muscular strength & endurance) +	NR	NR	Fewer opportunities for after-school PA programs -	NR
Stanish et al (2017)	NR	NR	NR	Lower activity time on weekends -	NR
Haegele et al (2018)	Lower physical fitness test passing rates -	NR	NR	NR	NR
Chu et al (2019)	Age -; Better motor skills (MC & SA) +	NR	NR	NR	NR
Garcia-Pastor et al. (2019)	Age -; Overweight/obesity -	NR	NR	NR	NR

Note: NR = not reported; + = positive association; - = negative association; **00** = inconsistent association; MC = manual coordination; SA = strength and agility; WASO = wake after sleep onset.

**Fig. 3:**
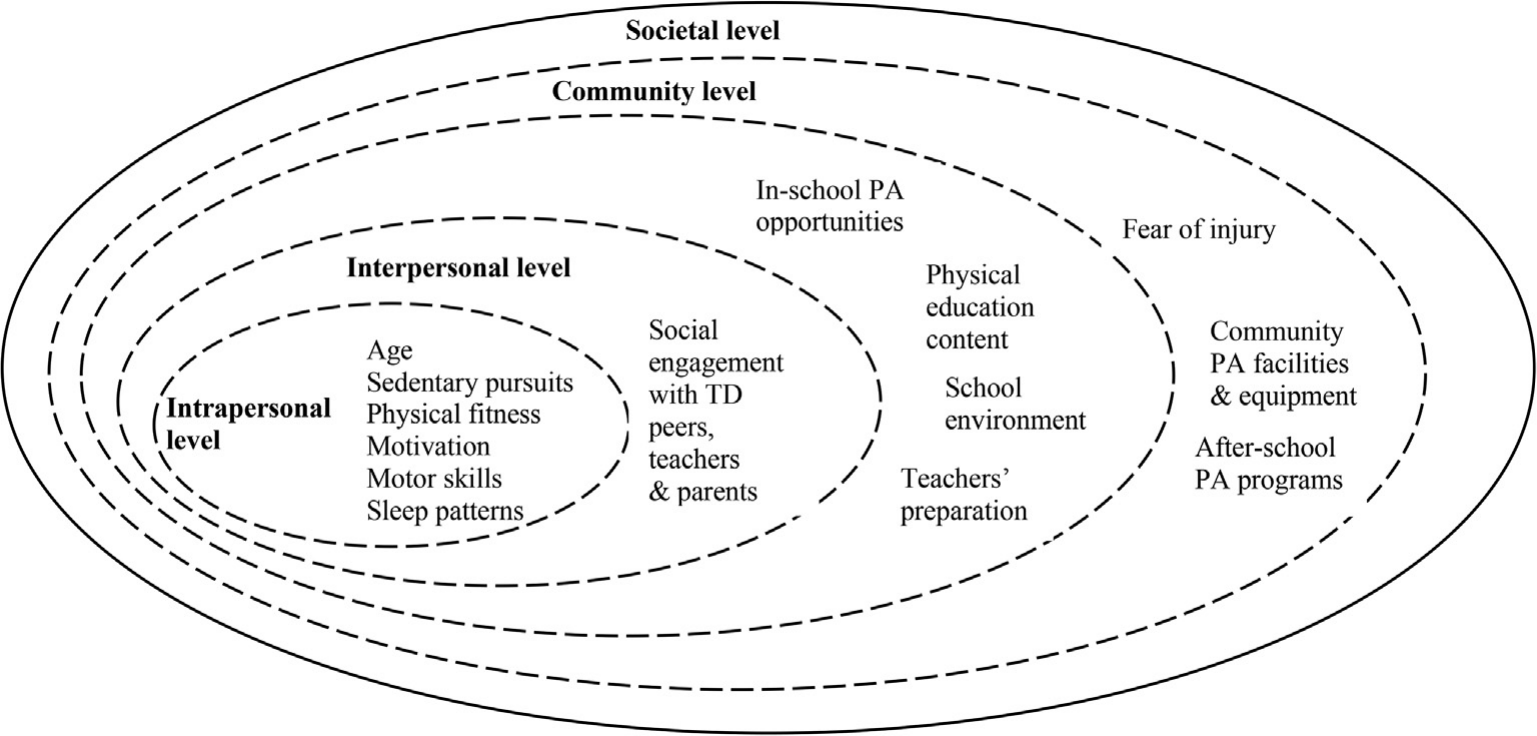
Factors affecting the PA levels of children and adolescents with ASD within the Social Ecological Model (SEM).

At the individual level, age was considered a critical factor affecting PA levels, with a younger age being associated with higher PA (Pan and Frey, 2005, Pan and Frey, 2006, Pan, 2009, MacDonald et al., 2011, Pan et al., 2011c, Wachob and Lorenzi, 2015, Chu et al., 2019, Garcia-Pastor et al., 2019). In addition, sedentary pursuits, physical fitness, self-determined motivation, motor skills, and sleep patterns affected their PA levels (Pan and Frey, 2005, Sandt and Frey, 2005, Obrusnikova and Cavalier, 2011, Pan et al., 2011b, Tyler et al., 2014, Wachob and Lorenzi, 2015, Haegele et al., 2018, Chu et al., 2019). Higher levels of fitness or motor performance, better motivation and sleep, and less sedentary behavior were related to higher PA. At the interpersonal level, having social support from TD peers, teachers, and parents was associated with higher PA (Pan, 2009, Obrusnikova and Cavalier, 2011, Pan et al., 2011a, Ayvazoglu et al., 2015). At the organisational level, school environment shortages (facilities & equipment), unprepared physical education content and a lack of teachers’ knowledge of teaching students with ASD, and limited PA opportunities in schools had a negative influence on the PA levels of children and adolescents with ASD (Sandt and Frey, 2005, Pan and Frey, 2006, Pan, 2008a, Pan, 2008b, Obrusnikova and Cavalier, 2011, Pan et al., 2015, Pan et al., 2011a). At the community level, the lack of after-school PA opportunities and negative experiences of community PA participation were associated with lower PA at weekends than on weekdays (Sandt and Frey, 2005, Obrusnikova and Cavalier, 2011, MacDonald et al., 2011, Bandini et al., 2012, Ayvazoglu et al., 2015, Pan et al., 2016, Stanish et al., 2017). At the societal level, no studies appear to have focused on the policies, laws, and regulations that affected the PA levels of children and adolescents with ASD. Multi-level factors that affected the PA levels of children and adolescents with ASD are summarized from the included studies in Fig. 3.

## 4 Discussion

The aim of this review was to determine the PA levels of children and adolescents with ASD and to identify the factors that facilitated or inhibited their PA levels, using the SEM. In general, the PA levels of children and adolescents with ASD on weekdays were slightly higher than at weekends (56.95 min of MVPA during weekdays and 55.72 min of MVPA during weekends). A previous review indicated that the estimated MVPA of children with ASD was 34–166 min/day (Jones et al., 2017), and our own results (56.95 min/weekdays vs. 55.72 min/weekends) fell within this range. We found that only 42% of participants in the included studies achieved the PAG of 60-min MVPA daily. We also found that 17% of participants in the included studies met the PAG for physical education lessons and 16.7% reached the PAG for recess time. Although no specific PAG exists for children and adolescents with disabilities, the *Global PAG for Health* recommends that, whenever possible, children and adolescents with disabilities should meet any health guidelines to achieve health benefits (World Health Organization, 2010).

The accelerometer has been widely used to assess objective PA data for children and adolescents with and without ASD (Pan et al., 2015). However, different protocols (e.g. wearing time, reporting methods, reported PA categories) were used to measure their PA levels in this review. Standard approaches to measuring their PA levels are recommended in order to improve the quality of data collection and interpretation of results for comparisons (Leung et al., 2017). Meanwhile, using a combination of PA measures is desirable as they can improve the understanding of the contextual factors that affect their PA levels (Capio et al., 2011). We found that only one study used both accelerometers and the BEACHES observation system (Sandt and Frey, 2005), while three studies used questionnaires and interviews (Bandini et al., 2012, Ayvazoglu et al., 2015, Stanish et al., 2017).

By applying the SEM, *at the individual level*, we found that PA in general declined with age. As children become older, game rules and required motor skills become more complex and children with ASD may not be able to adapt to competitive group games with their TD peers (Arnell et al., 2018). Meanwhile, engagement with electronic screen activities after school and at weekends was associated with decreased PA (Obrusnikova and Miccinello, 2012, Must et al., 2015). Sleep was also found to be associated with MVPA (Wachob and Lorenzi, 2015). It was supported by a recent study which indicated that children with ASD showed a significant improvement in sleep quality after a 12-week physical activity intervention (Tse et al., 2019). One study focused on the associations between motivation and PA levels in adolescents with ASD and found that external regulation was positively and significantly related to the time spent on MVPA during an inclusive physical education class (Pan et al., 2011b). One possible explanation is that adolescents with ASD are afraid of being excluded by their friends and TD peers (external regulation) during an inclusive physical education class.

*At the interpersonal level*, social engagement with TD peers, parents, and teachers are considered to be contributing factors for acquiring MVPA (Pan, 2009, Memari et al., 2017). Parents, teachers, and peers play a significant role in encouraging children and adolescents with ASD to engage in PA with TD peers (Arnell et al., 2018). A recent review found that adolescents with ASD reported that they were socially isolated and preferred solitary activities (e.g., watching TV, playing computer games, running) (Krieger et al., 2018). However, parents of children with ASD reported that their children experienced peer-related bullying at school and during community PA activities (Bandini et al., 2012, Ayvazoglu et al., 2015). Anti-bullying policies at the school level are needed to support participation in different activities for children and adolescents with ASD (Saggers et al., 2011).

*At the organizational level*, schools are the salient institutions where students with disabilities can accumulate MVPA on a daily basis (Sit et al., 2017). However, we found that children and adolescents with ASD spent only an average of 14.85 min in MVPA during physical education class, 16.75 min during recess, and 31.58 min after school. In-school tailor-made PA interventions should therefore be designed to promote PA levels of students with ASD to meet the PAG for physical education lessons and recess time. Individual education plans, cross-functional teamwork, available space and facilities, and student-oriented instruction should be implemented within the school-based curriculum to promote PA participation by all students (Sit et al., 2007; 2017). Other school settings such as lunchtime are also important in promoting activity accrual in students with disabilities (Sit et al., 2017).

*At the community level*, we found that parents of children and adolescents with ASD reported barriers to participation in community PA programs, including unwelcome attention and social isolation in the community, limited types of PA programs provided by communities, and financial challenges (transportation and cost of childcare) (Obrusnikova and Cavalier, 2011, Ayvazoglu et al., 2015). Consistent with our findings, Srinivasan et al. (2014) confirmed that parents of children with ASD had to pay for medical and behavioral treatment lasting 30 to 40 h per week, creating a heavy financial burden and limiting the extent to which children with ASD are able to participate in community PA. Must et al. (2015) also reported that the community-based PA opportunities for children with ASD were scarce and expensive.

*At the societal level*, we found that very few previous studies had examined the effects of written policies on PA levels in children and adolescents with ASD. No specific policies promote PA participation in the mainstream schools of students with disabilities (Qi et al., 2017). We therefore recommend that schools should be encouraged to formulate written policies at the school level, because clear and mandatory policies for physical education and recess not only help students with disabilities engage in daily PA, but also help them maintain their health and improve their quality of life into adulthood (Ridgers et al., 2012). For example, in China, the ‘Health China Initiative 2030’ policy introduced by the Chinese government requires primary and secondary students to exercise for two hours each day, including one hour of in-school PA and one hour after-school PA (The State Council of the People's Republic of China, 2019). Adopting clear and feasible written policies of time allocation for PA might increase the amount of daily PA engagement (Ridgers et al., 2012).

This is the first systematic review to examine the PA levels of children and adolescents with ASD, using PAGs and identifying the PA-related factors grounded with SEM as a theoretical framework. Overall, children and adolescents with ASD were physically inactive, and multi-level factors affecting their PA engagement were identified. The review has several limitations. First, the included studies had multiple methodological differences, including sample size, PA measuring tools, PAG, and statistical analysis, thereby making it difficult to synthesize the results for a meta-analysis. Second, most included papers utilized convenient sampling and did not recruit TD peers for comparison, which may have caused sampling bias. Third, several studies only included male adolescents with ASD as participants, so we could not tell whether gender was associated with PA in this population. Furthermore, there was a wide age range of participants in the included studies. It is evident that children’s PA changes from childhood to adolescence and we were unable to identify different PA-related factors for a specific age range.

## 5 Conclusion

This review shows that children and adolescents with ASD are insufficiently active to meet the various PAGs, and that the factors affecting their PA engagement are multifaceted. This review improves our understanding of objectively-measured PA in children and adolescents with ASD and the PA-related factors ranging from the individual to the societal level. Effective PA interventions should be designed and implemented to facilitate their activity accrual during and outside of school time. Future studies are needed to further explore the contextual factors and policies that affect their PA at different levels within the SEM.

## Declaration of Competing Interest

The authors declare that they have no known competing financial interests or personal relationships that could have appeared to influence the work reported in this paper.
